# Differences between Patterns of Brain Activity Associated with Semantics and Those Linked with Phonological Processing Diminish with Age

**DOI:** 10.1371/journal.pone.0099710

**Published:** 2014-06-27

**Authors:** Ruben Martins, France Simard, Oury Monchi

**Affiliations:** 1 Centre de Recherche, Institut Universitaire de Gériatrie de Montréal, Montréal, Canada; 2 Department of Radiologie, Faculté de Médecine, Université de Montréal, Montréal, Canada; 3 Department of Kinanthropology, Université du Québec à Montréal, Montréal, Canada; University of Udine, Italy

## Abstract

It is widely believed that language function tends to show little age-related performance decline. Indeed, some older individuals seem to use compensatory mechanisms to maintain a high level of performance when submitted to lexical tasks. However, how these mechanisms affect cortical and subcortical activity during semantic and phonological processing has not been extensively explored. The purpose of this study was to look at the effect of healthy aging on cortico-subcortical routes related to semantic and phonological processing using a lexical analogue of the Wisconsin Cart-Sorting Task. Our results indicate that while young adults tend to show increased activity in the ventrolateral prefrontal cortex, the dorsolateral prefrontal cortex, the fusiform gyrus, the ventral temporal lobe and the caudate nucleus during semantic decisions and in the posterior Broca's area (area 44), the temporal lobe (area 37), the temporoparietal junction (area 40) and the motor cortical regions during phonological decisions, older individuals showed increased activity in the dorsolateral prefrontal cortex and motor cortical regions during both semantic and phonological decisions. Furthermore, when semantic and phonological decisions were contrasted with each other, younger individuals showed significant brain activity differences in several regions while older individuals did not. Therefore, in older individuals, the semantic and phonological routes seem to merge into a single pathway. These findings represent most probably neural reserve/compensation mechanisms, characterized by a decrease in specificity, on which the elderly rely to maintain an adequate level of performance.

## Introduction

How does age-related cognitive decline affect language? Surprisingly, not many studies have tried to answer this question. One possible reason is that it is somewhat difficult to separate pure language processes from working memory, which is often required during the execution of language tasks [1] [2] [3] [4] [5]. Nonetheless, most existing studies indicate that language function shows little performance decline with healthy aging [6] [7] [8]even if some older individuals do show impaired execution during language production tasks [9], and that errors accessing phonological word forms tend to occur more often in the elderly [10]. Indeed, most of these findings can be explained by a decline in working memory instead of language per se [7]. Furthermore, some language attributes such as semantic knowledge clearly increase as time passes by [8] [11]. Therefore, normal aging is characterized by language abilities preservation despite important cerebral tissue loss including white matter integrity [12] [13] [14].

Syntactic and narrative discourse processing studies have reported that the elderly tend to show increased bilateral cerebral activity compared to younger individuals [15]. Because such patterns seem to be associated with preserved language function in the elderly [15], they have been postulated to reflect a compensatory mechanism similar to the HAROLD (hemispheric asymmetry reduction in older adults) model conceptualized by Cabeza [16]. Other neuroimaging studies that looked at language function have also reported increased bilateral activity in high performing older persons during verbal generation [17] and naming tasks [18]. More recently, Obler and colleagues [19] have even shown anatomical evidence (using diffusion tensor imaging) that older individuals with high naming skills tend to rely more extensively on right-hemisphere frontal regions (peri-Sylvian and the midfrontal areas). Therefore, language function has been proposed to depend on similar compensatory mechanisms as other cognitive processes to maintain high performance despite age-related atrophy. Those mechanisms are namely the mentioned HAROLD model and intrahemispheric reorganization of activation, mainly from the occipitotemporal to the frontal cortex [20] [21] [22] [23] [24], an observation referred by Dennis and Cabeza [25] as the Posterior-Anterior Shift in Aging (PASA) phenomenon. Indeed, Grossman et al. [26] showed that during the execution of a language comprehension task, older good “performers” showed increased prefrontal cortex (PFC) activity compared to younger participants. On the other hand, some semantic neuroimaging studies showed the opposite: older participants presented increased posterior activation compared to the young [27] [28] [29]. All of these findings, however, represent brain activity reorganization and can therefore be considered neural compensation mechanisms.

The elderly may also rely on pre-existing brain networks that are more efficient and less susceptible to age-induced disruption in order to maintain high levels of performance, a compensatory mechanism known as neural reserve [30]. Grossman et al. [26] have shown that, when both older good and poor “performers” were compared during the execution of a sentence-comprehension task, poor “performers” engaged significantly less activation in some important sentence-processing areas (inferior frontal cortex and posterior-superior temporal cortex) relatively to good “performers”. This finding appears to show that old good “performers” are able to rely more extensively on some well-preserved language networks, therefore using neural reserve as a compensatory mechanism.

In the present study, we aimed to explore how healthy aging affects two language functions, namely semantic and phonological processing. To do so we used the Wisconsin Word Sorting Task (WWST), a lexical analog of the Wisconsin Card Sorting Task (WCST) [31]. The principles governing this task are exactly the same as those in the original WCST. However, in the WWST, subjects have to classify words, instead of pictograms, according to one of the three following lexical rules: semantic, syllable onset, or syllable rhyme. In particular, the present study was designed to explore the compensatory mechanisms on which high performing older individuals rely to preserve language abilities and what is the specific effect of those mechanisms on the cortico-subcortical routes related to semantic and phonological processing.

We hypothesized that network specificity would be reduced with aging. Indeed, we thought that the elderly, in order to maintain performance, would rely extensively on neural reserve and compensation for both semantic and phonological processing which would lead to a loss in network specificity between rules [30]. We also expected reaction times to follow the same pattern as cerebral activity and therefore show fewer differences between classification rules in the older compared with the younger group since previous studies have shown that brain activity and reaction times were correlated [1]. Furthermore, given the fact that young healthy candidates have shown important frontal and bilateral activity during the performance of the WWST [32], we wanted to explore what would happen with respect to reduced hemispheric asymmetry (HAROLD model) and intrahemispheric reorganization of activity (PASA phenomenon) in the older group when compared to the younger one.

## Materials and Methods

### Subjects

Twenty-height French-speaking right-handed subjects (fourteen [younger group] whose mean age was 26 [range ±5; 8 females, 6 males], and fourteen [older group] whose mean age was 63 [range: ±8.0; 6 females, 8 males]) with no personal nor familial history of psychiatric or neurological disorder participated in the present study. Handedness was assessed by the Edinburgh Handedness Inventory. Participants gave written informed consent to the protocol, which was been approved by the research ethics committee of the Regroupement Neuroimagerie Québec (CMER-RNQ). This committee follows the guidelines of the civil code of Quebec, the Tri-Council Policy Statement of Canada, the Declaration of Helsinki, and the code of Nuremberg.

### Cognitive task

The Wisconsin Word Sorting Task (WWST) developed by Simard et al. [31] was administered using a stimulus presentation software named Media Control Function (Digivox, Montréal, Canada). This task has been previously used to study executive functions such as planning and set-shifting in both young [31] and older adults [33]. However, it can also be used to explore semantic and phonological processes by analysing accurate word matching according to semantics and to syllable onset/rhyme respectively [32]. The WWST is a lexical analog of the computerized Wisconsin Card Sorting Task (WCST) developed by Monchi et al. [34]; however, instead of using pictogram cards, it uses French words. A strict correspondence regarding the stimuli, the rules, and the number of exemplars was established between the two tasks [31]. Explicitly, the three classification rules of the WCST (i.e. classification according to color, shape, and number of visual stimuli) were replaced by three lexical ones: one semantic and two phonological rules, syllable onset (attack) and syllable rhyme.

Throughout the task, a new test word was shown in the middle of the screen below a reference row composed of four fixed words: bateau (ship), araignée (spider), cadran (clock) and poivron (pepper). During scanning, the computer display was projected onto a mirror in the MRI scanner. On each trial, participants had to match the test word with one of the reference words based on (1) semantic categorization, (2) syllable rhyme or (3) syllable onset. To select a word, subjects had to press the appropriate buttons of a magnetic resonance imaging compatible response box held with their right hand: the left button moved a cursor under the reference card from left to right, while the selection was made by pressing the right button. On each trial, participants had to find the proper classification rule and apply it based on the feedback he/she received following each selection. A change in the screen brightness indicated whether the answer was correct (bright screen) or not (dark screen). After six consecutive correct trials, the classification rule changed without warning and the subject had to discover the new appropriate rule.

Similarly to the original WCST, there were four matching possibilities for each one of the categories in the WWST: 4 semantic: transportation, animals, objects, and vegetables; 4 phonological onset syllables: ‘ba’, ‘a’, ‘ca’, ‘poi’; and 4 phonological rhyme syllables: ‘au’, ‘é’, ‘an’, ‘on’. The words have been all carefully chosen so they could have the same phonological syllabic structure and be considered concrete according respectively to the French lexical database *lexique 3* [35] and the concreteness scale of Bonin et al. [36]. Words were four to nine letters long with either two to three syllables. Firstly, the words that shared the most onset and rhyme syllables were chosen and matched into four categories. Then, from this selection, the words that shared the same semantic category were selected.

The WWST trials contained two sorts of periods: a matching period and a feedback period. The matching started with the presentation of a new test word and continued until reference word selection. The length of this period varied from trial to trial depending on participant's response time. Matching periods were followed by a feedback period, which lasted 2.3 sec and started as soon as a selection was made. Positive feedbacks were indicated by a bright screen and informed the subject that the current classification rule was the correct one, while negative feedbacks were indicated by a dark screen and informed the participant that the selection was incorrect and therefore a shift was required. These periods ended with the presentation of a new test word on the screen initiating a new trial.

Every subject participated in one fMRI session. Each scanning session contained four functional runs; each of one was made up of four task blocks. All blocks consisted of three experimental (corresponding to each one of the three rules) and one control condition presented in a pseudo-random fashion. Just before the scanning session began, subjects were fully trained on the task using a personal computer. Everyone of them practiced until their performance reached a time response plateau with less than 6% of perseverative (incorrect use of the same classification rule following negative feedback more than twice in a row) and non-perseverative (the participant incorrectly changes the classification rule after having correctly applied it at least three times) errors. Finally, prior to training, participants were also familiarized with the list of test words in order to verify that they knew all of them and could classify each one within one of the four semantic categories. For the present study, we explored exclusively language processing, therefore we focused solely on the successful matching periods following positive feedback (removing the first positive trial after a set-shift or an error). Nine contrasts were generated for statistical analysis by subtracting the control matching period trials from that of the matching following positive feedback periods for each of the three classification rules as well as by subtracting the matching following positive feedback period trials of one rule from the same period of another rule. Explicitly, these contrasts are (1) matching following positive feedback according to semantics minus control matching; (2) matching following positive feedback according syllable onset to minus control matching; (3) matching following positive feedback according to rhyme minus control matching; (4) matching following positive feedback according to semantics minus matching following positive feedback according to syllable onset; (5) matching following positive feedback according to semantics minus matching following positive feedback according to rhyme; (6) matching following positive feedback according to syllable onset minus matching following positive feedback according to semantics; (7) matching following positive feedback according to syllable onset minus matching following positive feedback according to rhyme; (8) matching following positive feedback according to rhyme minus matching following positive feedback according to semantics; (9) matching following positive feedback according to rhyme minus matching following positive feedback according to syllable onset.

### fMRI scanning

Every participant was scanned at the Unité de Neuroimagerie Fonctionnelle of the Institut de Gériatrie de Montréal using a 3T Siemens TIM MRI scanner (Siemens AG, Erlangen, Germany). Scanning sessions began with a high-resolution T1-weighted three-dimensional volume acquisition for anatomical localization (voxel size, 1×1×1 mm^3^), followed by echoplanar T2*-weighted images with BOLD contrast (TE, 30 msec; FA, 90°) acquisitions. Functional images were acquired every 2.5 sec in four runs containing 210 volumes, and each volumes contained 36 slices with a matrix size 64×64 pixels (voxel size, 3.5×3.5×3.5 mm^3^). Stimuli presentation and scanning were synchronized at the beginning of each run.

It should be noted that the whole data linked to this study can be made available on a secured server upon request to the corresponding author.

### Data analysis

The fMRI data was analyzed following the same method as in our previous studies [31] [32] [33] [34] [37]. It made use of the fMRIstat software developed by Worsley et al. [38]. For the analysis, the first three frames in each run were discarded. Images from all runs were first realigned to the fourth frame for motion correction and smoothed using a 6 mm full width half-maximum (FWHM) isotropic Gaussian kernel. The statistical analysis of the fMRI data was based on a linear model with correlated errors. The design matrix of the linear model was first convolved with a difference of two gamma hemodynamic response functions timed to coincide with the acquisition of each slice. Furthermore, the correlation structure was modelled as an autoregressive process. At each voxel, after bias correction for correlation induced by the linear model, the autocorrelation parameter was estimated from the least square residuals. The autocorrelation parameter was first regularized by spatial smoothing and was then used to ‘whiten’ the data and the design matrix. The linear model was re-estimated using least squares on the whitened data to produce estimates of effects and their standard errors. Then, the resulting effects and standard effect files were spatially normalized by non-linear transformation into the MNI 305 standard proportional stereotaxic space, which is based on that of Talairach and Tournoux [39], using the algorithm of Collins and colleagues [40]. Anatomical images were also normalized using the same space and transformation. In a second step, using a mixed effects linear model for the data taken from the previous analysis, runs and subjects were combined. A random effects analysis was performed by first estimating the ratio of the random effects variance to the fixed effects variance, then regularizing this ratio by spatial smoothing with a Gaussian filter. Inter-group analyses were performed by direct comparisons using the effects and standard deviations files of all individuals from both groups. The amount of smoothing was chosen so that 100 effective degrees of freedom would be achieved [38] [41]. Statistical maps were thresholded at p<0.05 correcting for multiple comparisons using the minimum between a Bonferroni correction as well as random field theory in the single and inter-group analysis. This yields a threshold of t>4.70 for a single voxel or a cluster size >534 mm^3^ for a significance assessed on the special extent of contiguous voxel [42]. Peaks within the basal ganglia, thalamus, and PFC that were observed in our previous studies using the WWST in young healthy adults [32] were considered predicted and are reported at a significance of p<0.001 uncorrected [indicated by an asterisk (*) in the tables].

Behavioral data (reaction times) were also collected, and intra and inter-group analyses were performed using SPSS 15.0 for Windows. A comparison between the two groups for each classification rule and between classification rules for each group was performed using T-Tests and ANOVAs. For these analyses, the reaction times for control matching trials were subtracted from those of the classification rules in order to account for age-related motor-speed decline [43] [44].

## Results

### Behavioral performance

On average, in the younger group, control matching lasted 1285 msec (±166 msec), matching following positive feedback according to semantics lasted 1785 msec (±235 msec), matching according to syllable onset lasted 1531 msec (±198 msec) and matching following according to syllable rhyme lasted 1695 msec (±181 msec). When removing control matching from the different matching following positive feedback periods, then matching according to semantics only lasted 500 msec (±144 msec), matching according to syllable onset lasted 246 msec (±128 msec) and matching according to syllable rhyme lasted 410 msec (±149 msec).

In the older group, control matching lasted 1795 msec (±292 msec), matching following positive feedback according to semantics lasted 2357 msec (±495 msec), matching according to syllable onset lasted 2282 msec (±534 msec) and matching according to syllable rhyme lasted 2399 msec (±538 msec). However, by removing control matching from matching following positive feedback, matching according to semantics only lasted 562 msec (±273 msec), matching according to syllable onset lasted 487 msec (±278 msec) and matching according to syllable rhyme lasted 604 msec (±285 msec).

Older individuals proved to be slower than younger ones for all conditions (Control: p<0.001 t = 5.446, semantics: p = 0.001 t = 3.790, syllable onset: p<0.001 t = 4.851, syllable rhyme: p<0.001 t = 4.581). However, only time responses during phonological rules proved to be slower in the older group when control times were subtracted (Semantics: p = 0.4765 t = 0.724, syllable onset: p = 0.009 t = 2.864, syllable rhyme: p = 0.041 t = 2.176).

When we perform comparisons between rules within each group, we find that, for younger individuals, response times tend to be shorter for syllable onset compared both to semantics and to syllable rhyme taking or not control response times into account (ANOVA: F = 5.477, p = 0.008 [semantics vs syllable onset: p = 0.005 t = 3.093, syllable rhyme vs. syllable onset: p = 0.031 t = 2.287]; ANOVA - control response times subtracted – F = 11.743, p<0.001 [semantics vs syllable onset: p<0.001 t = 4.933, syllable rhyme vs. syllable onset: p = 0.004, t = 3.124]). On the other hand, all these differences disappear for the older group (ANOVA: F = 0.129 p = 0.880;ANOVA - control response times subtracted – F = 0.452, p = 0.641).

### fMRI results

As predicted, the analysis revealed that differences between semantic and phonological pathways tend to diminish with aging. Indeed, while younger individuals showed increased ventrolateral PFC activity during matching according to semantics compared to matching according to syllable onset or rhyme, older individuals did not. Also, when matching according to one of the phonological rules was compared to matching according to semantics, younger individuals showed increased posterior prefrontal activity (rhyme) and posterior parietal activity (onset), while older individuals, once more, did not show increased activity at all.

All significant activation for the younger adults, the older adults and intergroup comparisons are reported in this section. Tables 1 to 9 contain a complete description of all regions significantly activated for younger and older adults as well as intergroup contrasts. The complete results for the younger group can also be found in the study by Simard et al.[32].

**Table 1:** Matching according to semantics minus control matching in the YOUNG.

Anatomical area	Hemisphere	Stereotaxic coordinates	T stat	Cluster size
**YOUNG**				
Frontopolar cortex (area 10)	Left	−42 54 −4	7.22	>10000
Anterior cingulate cortex (area 32)	Left	−8 36 28	3.72	>10000
	Right	10 36 28	5.33	>10000
Ventrolateral prefrontal cortex (area 47/12)	Left	−30 30 4	7.46	>10000
	Right	36 28 0	6.62	3120
Ventrolateral prefrontal cortex (area 45)	Left	−48 28 20	6.95	>10000
Dorsolateral prefrontal cortex (areas 9, 9/46)	Left	−52 28 28	7.22	>10000
Superior frontal cortex (area 6, 8 SMA)	Left	−4 20 50	8.31	>10000
Lateral premotor cortex (area 6)	Left	−48 8 44	5.66	>10000
Posterior cingulate cortex (area 23)	Left	−2 −34 26	4.9	2520
Inferior temporal cortex (area 37)	Left	−46 −62 −6	6.15	>10000
Lateral posterior parietal cortex (area 7)	Left	−26 −62 42	7.5	>10000
	Right	28 −68 52	4.41	>10000
Occipital cortex (area 19)	Left	−30 −70 −10	6.79	>10000
	Right	22-68 8	7.08	>10000
Occipital cortex (area 18)	Left	−38 −80 −8	7.84	>10000
	Right	30 −86 4	7.51	>10000
Occipital cortex (area 17)	Left	−8 −84 4	7.15	>10000
	Right	8 −84 8	8.55	>10000
Thalamus	Left	−6 −14 10	5.32	5272
	Left	−26 −34 6	5.09	>10000
	Right	22 −28 0	5.47	824
	Right	8 −14 10	3.87	5272
Caudate nucleus (head)	Left	−12 −2 16	3.58	5272
	Right	12 8 2	4.05	5272
Cerebellum	Left	−38 −62 −28	4.07	>10000
	Right	34 −74 −18	6.45	>10000
**YOUNG VS OLD**				
Anterior cingulate cortex (area 32)	Left	−16 28 2	3.78	280
	Right	12 32 22	4.12	520
Ventrolateral prefrontal cortex (area 45)	Left	−48 22 20	5.19	7424
Dorsolateral prefrontal cortex (area 9)	Left	−56 24 28	4.88	7424
Superior frontal cortex (area 6, 8 SMA)	Left	−4 12 54	4.17	2152
Lateral premotor cortex (area 6)	Left	−48 2 40	4.35	736
Posterior cingulate cortex (areas 23, 31)	Left-area 31	−20 −66 8	5.30	>10000
	Left-area 23	−2 −26 30	4.71	1128
	Right-area 31	8 −68 14	6.28	>10000
Lateral posterior parietal cortex (area 7)	Left	−32 −56 58	4.00	>10000
Precuneus (area 7)	Left	−10 −66 50	6.25	>10000
	Right	4 −86 42	5.14	>10000
Occipital cortex (area 19)	Left	−24 −66 38	6.47	>10000
	Right	8 −82 40	4.56	>10000
Occipital cortex (area 18)	Left	−30 −84 12	6.37	>10000
	Right	8 −86 8	6.40	>10000
	Right	34 −86 4	5.49	>10000
Occipital cortex (area 17)	Left	−1 −74 12	6.03	>10000
	Right	8 −88 6	6.45	>10000
Thalamus	Left	−20 −32 0	5.25	1792
	Right	22 −30 0	5.26	1072
Cerebellum	Left	−16 −86 −16	6.04	>10000

**Table 2:** Matching according to semantics minus control matching in the OLD.

Anatomical area	Hemisphere	Stereotaxic coordinates	T stat	Cluster size
**OLD**				
Anterior cingulate cortex (area 32)	Left	−8 30 40	3.98	936
Dorsolateral prefrontal cortex (areas 9, 9/46)	Left	−50 26 30	3.49	336
Superior frontal cortex (area 6, 8 SMA)	Left	−2 10 68	4.11	328
Cerebellum	Right	12 −86 −30	3.63	256
**OLD VS YOUNG**				
Frontopolar cortex (area 10)	Left	−6 64 0	5.42	7848
	Right	4 60 −4	5.72	7848
Anterior cingulate cortex (area 32)	Left	−6 50 −4	4.51	7848
Insula (areas 41, 43)	Left -area 43	−40 0 2	4.80	3224
	Left -area41	−40 16 4	4.39	3224
	Right -area43	40 0 8	4.28	552
	Right -area 41	36 4 −16	4.22	272
Posterior inferior parietal cortex (area 40)	Left	−60 −30 22	5.33	2472
	Right	58 −30 22	4.69	3848
Middle Temporal Cortex (area 39)	Left	−50 −64 14	4.75	5912
	Right	42 −58 18	4.40	2664
Superior Temporal Cortex (area 22)	Left	−64 −54 16	3.95	5912
	Right	52 −56 16	4.14	2664
Posterior cingulated cortex (area 31)	Right	2 −50 36	4.02	1904
Occipital cortex (area 19)	Right	−44 −78 34	4.22	5912
Cerebellum	Left	−22 −84 −36	4.35	464

**Table 3:** Matching according to syllable onset minus control matching in the YOUNG.

Anatomical area	Hemisphere	Stereotaxic coordinates	T stat	Cluster size
**YOUNG**				
Frontopolar cortex (area 10)	Left	−38 62 8	9.91	5904
Anterior cingulate cortex (area 32)	Left	−2 42 34	3.53	>10000
	Right	8 34 32	3.5	>10000
Ventrolateral prefrontal cortex (area 47/12)	Left	−30 28 2	6.29	2120
	Right	32 28 0	5.1	1464
Ventrolateral prefrontal cortex (area 45)	Left	−48 28 20	5.55	>10000
Dorsolateral prefrontal cortex (areas 9, 9/46)	Left	−52 28 28	5.55	>10000
Superior frontal cortex (area 6, 8 SMA)	Left	−2 22 48	9.1	>10000
Lateral premotor cortex (area 6)	Left	−48 6 42	6.11	>10000
Posterior inferior parietal cortex (area 40)	Left	−34 −46 44	5.26	>10000
Inferior temporal cortex (area 37)	Left	−48 −62 −10	6.22	>10000
Lateral posterior parietal cortex (area 7)	Left	−26 −60 42	7.53	>10000
	Right	30 −64 50	5.26	>10000
Occipital cortex (area 19)	Left	−22 −90 22	4.94	>10000
	Right	22 −90 28	6.8	>10000
Occipital cortex (area 18)	Left	−20 −86 −10	7.77	>10000
	Right	8 −82 4	7.5	>10000
Occipital cortex (area 17)	Left	−16 −90 −6	6.85	>10000
	Right	18 −94 −8	5.68	>10000
Thalamus	Left	−6 −14 10	4.15	664
	Left	−22 −32 4	4.53	704
	Right	20 −30 14	4.02	640
Globus pallidus	Left	−16 0 8	3.58	664
	Right	14 0 4	4.73	984
Cerebellum	Left	−28 −66 −30	5.8	>10000
	Right	34 −74 −18	6.82	>10000
**YOUNG VS OLD**				
Ventrolateral prefrontal cortex (area 47/12)	Left	−30 28 6	3.80	192
Ventrolateral prefrontal cortex (area 45)	Left	−50 24 20	3.72	440
Dorsolateral prefrontal cortex (area 9)	Left	−52 22 24	3.83	440
Superior frontal cortex (area 6, 8 SMA)	Right	8 16 50	3.85	136
Posterior inferior parietal cortex (area 40)	Left	−22 −62 38	5.21	5624
	Left	−38 −46 46	4.44	984
Inferior temporal cortex (area 37)	Left	−44 −44 −10	3.88	488
Lateral posterior parietal cortex (area 7)	Left	−24 −66 50	5.51	5624
	Right	28 −68 50	3.80	940
Precuneus (area 7)	Left	−4 −78 54	3.50	224
	Right	4 −84 42	4.73	>10000
Posterior cingulate cortex (area 30)	Left	−20 −64 8	4.00	>10000
Occipital cortex (area 19)	Left	−22 −74 32	3.55	>10000
	Right	22 −88 28	4.62	1616
Occipital cortex (area 18)	Left	−16 −88 −12	4.98	>10000
	Left	−38 −80 −10	4.02	664
	Right	10 −72 16	5.31	>10000
Occipital cortex (area 17)	Left	−6 −72 12	4.23	>10000
	Right	6 −80 14	5.18	>10000
Cerebellum	Left	−4 −72 −36	4.06	328

**Table 4:** Matching according to syllable onset minus control matching in the OLD.

Anatomical area	Hemisphere	Stereotaxic coordinates	T stat	Cluster size
**OLD**				
Dorsolateral prefrontal cortex (areas 9, 9/46)	Left	−42 14 34	4.10	2168
Superior frontal cortex (area 6, 8 SMA)	Left	−4 36 40	3.94	792
Posterior inferior parietal cortex (area 40)	Left	−38 −52 38	3.92	1896
Lateral posterior parietal cortex (area 7)	Left	−32 −72 46	3.69	408
Occipital cortex (area 19)	Left	−30 −94 12	3.51	4560
	Right	32 −80 18	3.67	144
Occipital cortex (area 18)	Left	−30 −90 8	3.88	4560
	Right	12 −80 2	3.95	1456
Occipital cortex (area 17)	Left	−30 −94 −2	3.95	4560
	Left	−16 −88 18	3.59	1136
	Right	32 −86 −4	3.80	3040
Cerebellum	Right	−42 14 −20	4.02	3040
	Right	−28 −80 −10	3.95	4560
**OLD VS YOUNG**				
Frontopolar cortex (area 10)	Left	−4 70 4	4.17	2752
	Right	4 60 −4	4.10	2752
Anterior cingulate cortex (area 32)	Left	−1 22 −6	3.76	1888
Superior frontal cortex (area 6, 8 SMA)	Right	6 −12 70	4.04	160
Lateral premotor cortex (area 6)	Left	−40 −2 16	4.13	936
	Left	−52 −6 4	3.97	544
Insula (areas 41, 43)	Left –area 41	−40 −18 2	4.31	648
	Left –area 43	−54 −8 8	3.98	544
Posterior inferior parietal cortex (area 40)	Left	−58 −28 22	4.88	3480
	Right	60 −30 28	4.61	2952
Inferior temporal cortex (area 38)	Left	−36 4 −14	6.95	376
	Right	36 4 −16	4.15	736
Middle temporal cortex (area 39)	Left	−50 −72 14	4.03	1520
	Right	50 −56 12	3.80	1888
Superior temporal cortex (area 22)	Right	42 −56 20	4.69	1888
Precuneus (area 7)	Left	−8 −32 44	4.04	2168
	Right	2 −34 48	4.09	2168
Posterior cingulate cortex (area 31)	Left	−14 −30 40	4.63	2168
	Right	12 −24 44	4.56	304
Occipital cortex (area 19)	Left	−40 −78 40	4.20	744
Cerebellum	Left	−24 −84 −36	3.81	488

**Table 5:** Matching according to syllable rhyme minus control matching in the YOUNG.

Anatomical area	Hemisphere	Stereotaxic coordinates	T stat	Cluster size
**YOUNG**				
Frontopolar cortex (area 10)	Left	−26 54 14	4.27	1928
Anterior cingulate cortex (area 32)	Left	−8 30 36	4.48	>10000
	Right	8 36 30	4.54	>10000
Ventrolateral prefrontal cortex (area 47/12)	Left	−30 28 2	5.87	3016
	Right	32 28 0	5.36	1816
Ventrolateral prefrontal cortex (area 45)	Left	−48 28 20	5.71	>10000
Dorsolateral prefrontal cortex (area 9)	Left	−46 24 30	5.07	>10000
Posterior prefrontal cortex (area 44)	Left	−34 12 30	5.14	>10000
Superior frontal cortex (area 6, 8 SMA)	Left	−4 14 56	7.03	>10000
	Right	10 22 44	4.08	>10000
Lateral premotor cortex (area 6)	Left	−50 8 44	5.74	>10000
Posterior inferior parietal cortex (area 40)	Left	−28 −50 42	4.63	>10000
Inferior temporal cortex (area 37)	Left	−42 −62 −12	5.82	>10000
	Right	32 −54 −16	3.86	>10000
Lateral posterior parietal cortex (area 7)	Left	−24 −62 42	7.09	>10000
	Right	28 −66 42	4.4	>10000
Occipital cortex (area 19)	Left	−8 −84 8	6.14	>10000
	Right	18 −88 24	7.23	>10000
Occipital cortex (area 18)	Left	−18 −60 6	6.53	>10000
	Right	10 −72 16	8.96	>10000
Occipital cortex (area 17)	Left	−12 −70 12	6.79	>10000
	Right	12 −86 4	8.31	>10000
Thalamus	Left	−8 −12 10	5.29	2104
	Left	−22 −32 4	4.28	760
	Right	18 −14 14	4.7	4848
	Right	22 −28 0	5.95	4848
Globus pallidus	Right	12 −2 0	5.47	4848
Cerebellum	Left	−4 −66 −22	4.68	>10000
	Right	6 −76 −26	6.68	>10000
**YOUNG VS OLD**				
Dorsolateral prefrontal cortex (area 46)	Left	−48 24 20	3.86	136
Superior frontal cortex (area 6, 8 SMA)	Left	−50 2 40	3.76	144
Posterior inferior parietal cortex (area 40)	Left	−18 −62 54	4.11	2024
	Left	−24 −66 38	3.91	2024
Precuneus (area 7)	Right	4 −84 40	3.76	256
Occipital cortex (area 19)	Left	−6 −66 2	4.44	>10000
	Right	34 −86 4	5.21	>10000
Occipital cortex (area 18)	Left	−20 −64 8	4.74	>10000
	Right	12 −74 14	6.45	>10000
Occipital cortex (area 17)	Left	−12 −68 10	4.94	>10000
	Right	10 −66 12	6.13	>10000

**Table 6:** Matching according to syllable rhyme minus control matching in the OLD.

Anatomical area	Hemisphere	Stereotaxic coordinates	T stat	Cluster size
**OLD**				
Dorsolateral prefrontal cortex (areas 9)	Left	−38 6 34	3.88	952
Superior frontal cortex (area 6, 8 SMA)	Left	−4 18 52	4.10	1128
	Right	6 670	3.88	168
Occipital cortex (area 19)	Left	−6 −88 −12	3.63	200
	Right	32 −90 −4	3.62	352
Occipital cortex (area 18)	Right	16 −78 −16	4.10	3088
Occipital cortex (area 17)	Left	−4 −90 −10	3.56	200
	Right	14 −92 2	3.70	952
Cerebellum	Right	30 −66 −20	4.01	3088
**OLD VS YOUNG**				
Dorsolateral prefrontal cortex (area 46)	Right	44 38 10	4.62	920
Lateral premotor cortex (area 6)	Left	−38 2 12	4.48	6296
	Right	38 2 16	4.60	2144
Insula (areas 41, 43)	Left –area 41	−42 16 0	4.25	264
	Left –area 43	−52 −8 8	4.44	6296
	Right –area 43	40 −12 20	3.86	2144
Posterior inferior parietal cortex (area 40)	Left	−60 −28 22	5.89	6296
	Right	60 −30 26	5.31	7688
Inferior temporal cortex (area 38)	Left	−36 2 −14	4.57	456
Middle temporal cortex (area 39)	Left	−40 −76 34	3.64	352
	Right	50 −72 36	3.94	888
Superior temporal cortex (area 22)	Right	58 −34 20	4.67	7688
Occipital cortex (area 19)	Left	−6 −66 2	4.44	>10000
Cerebellum	Left	−22 −84 −38	3.83	320

**Table 7:** Matching according to semantic compared with matching according to syllable onset.

Anatomical area	Hemisphere	Stereotaxic coordinates	T stat	Cluster size
**YOUNG**				
**Semantics minus syllable onset**				
Mid-ventrolateral prefrontal cortex (area 47)	Left	−38 28 4	3.83*	352
Ventrolateral prefrontal cortex (area 45)	Left	−30 24 14	3.93*	344
Inferior temporal cortex (area 37, FG)	Left	−20 −48 −6	3.95*	128
Inferior temporal cortex (area 20)	Left	−42 −30 −20	3.36*	40
Occipital cortex (area 18)	Right	18 −94 14	4.17	936
Occipital cortex (area 17)	Right	4 −72 8	3.92	1448
**Syllable onset minus semantics**				
Frontopolar cortex (area 10)	Right	6 68 0	3.92*	208
Inferior parietal cortex (area 40)	Left	−60 −32 52	3.34*	80
	Right	44 −36 52	4.35*	360
Inferior temporal cortex (area 37, FG)	Left	−52 −64 −2	3.37*	48
**YOUNG VS OLD**				
**Semantics minus syllable onset**				
Occipital cortex (area 17)	Right	18 −92 6	3.90	528
Occipital cortex (area 18)	Right	6 −76 4	3.62	616
**Syllable onset minus semantics**				
-	-	-	-	-
**OLD**				
**Semantics minus syllable onset**				
-	-	-	-	-
**Syllable onset minus semantics**				
-	-	-	-	-
**OLD VS YOUNG**				
**Semantics minus syllable onset**				
-	-	-	-	-
**Syllable onset minus semantics**				
-	-	-	-	-

**Table 8:** Matching according to semantic compared with matching according to syllable rhyme.

Anatomical area	Hemisphere	Stereotaxic coordinates	T stat	Cluster size
**YOUNG**				
**Semantics minus syllable rhyme**				
Mid-ventrolateral prefrontal cortex (area 47)	Left	−56 30 −4	4.38	3656
Ventrolateral prefrontal cortex (area 45)	Left	−58 32 4	3.95	3656
Dorsolateral prefrontal cortex (area 9/46)	Left	−54 34 24	4.44	2016
Hippocampus (area 36)	Left	−30 −38 −12	4.11	392
Inferior temporal cortex (area 20)	Left	−38 −16 −24	3.28*	16
Occipital cortex (area 17)	Left	−14 −94 0	4.28	1560
Caudate nucleus	Right	16 14 2	3.3*	24
**Syllable rhyme minus semantics**				
Posterior prefrontal cortex (area 44)	Left	−41 3 20	3.43*	32
Inferior temporal cortex (area 20)	Right	48 0 −40	4.09*	208
Inferior temporal cortex (area 37, FG)	Left	−46 −66 −2	3.57*	136
Occipital cortex (area 17)	Right	18 −90 6	3.6*	192
**YOUNG VS OLD**				
**Semantics minus syllable rhyme**				
Ventrolateral prefrontal cortex (area 47/12)	Left	−30 26 −2	4.10	504
Posterior cingulate cortex (area 23)	Left	−2 −34 26	4.04	504
Inferior temporal cortex (area 20)	Left	−36 −44 −18	3.88	320
Inferior parietal cortex (area 40)	Left	−34 −70 38	3.56	352
Precuneus (area 7)	Left	−8 −68 50	3.95	464
Occipital cortex (area 17)	Left	−16 −84 −10	4.72	2464
**Syllable rhyme minus semantics**				
-	-	-	-	-
**OLD**				
**Semantics minus syllable rhyme**				
-	-	-	-	-
**Syllable rhyme minus semantics**				
-	-	-	-	-
**OLD VS YOUNG**				
**Semantics minus syllable rhyme**				
-	-	-	-	-
**Syllable rhyme minus semantics**				
Superior frontal cortex (area 6, 8 SMA)	Left	−4 16 52	3.69	224
Posterior cingulate cortex (area 23)	Left	−2 −32 26	4.05	504
Precuneus (area 7)	Left	−8 −68 50	4.02	312
Occipital cortex (area 17)	Left	−12 −94 2	4.21	3144
Occipital cortex (area 18)	Left	−14 −88 −10	4.82	3144
Occipital cortex (area 19)	Left	−18 −84 −10	4.40	3144
Cerebellum	Left	−28 −74 −14	3.68	264

**Table 9:** Matching according to syllable onset compared with matching according to syllable rhyme.

Anatomical area	Hemisphere	Stereotaxic coordinates	T stat	Cluster size
**YOUNG**				
**Syllableonset minus syllablerhyme**				
Inferior parietal cortex (area 40)	Right	34 −40 48	4.61	960
Occipital cortex (area 18)	Left	−16 −88 −6	5.17	1304
**Syllablerhyme minus syllableonset**				
Anterior cingulate cortex (area 32)	Right	12 38 20	3.84*	240
Occipital cortex (area 17)	Left	−8 −72 18	4.63	>10000
	Right	10 −68 10	5.54	>10000
Occipital cortex (area 18)	Left	−12 −62 6	3.96	>10000
	Right	18 −86 −2	6.54	>10000
Occipital cortex (area 19)	Left	−18 −54 −6	4.56	>10000
**YOUNG VS OLD**				
**Syllableonset minus syllablerhyme**				
Mid-dorsolateral prefrontal cortex (area 9)	Right	42 28 42	4.04	408
Inferior parietal cortex (area 40)	Right	32 −40 46	4.10	640
Occipital cortex (area 17)	Left	−14 −90 −8	4.70	1296
Occipital cortex (area 18)	Left	−16 −88 −8	4.70	1296
**Syllablerhyme minus syllableonset**				
Occipital cortex (area 17)	Right	18 −92 6	4.90	2144
	Right	16 −72 12	4.07	2328
Occipital cortex (area 18)	Right	22 −88 −4	5.03	2144
**OLD**				
**Syllableonset minus syllablerhyme**				
-	-	-	-	-
**Syllablerhyme minus syllableonset**				
-	-	-	-	-
**OLD VS YOUNG**				
**Syllableonset minus syllablerhyme**				
Occipital cortex (area 17)	Right	18 −88 −4	5.46	3408
Occipital cortex (area 18)	Right	22 −88 −4	5.46	3408
	Right	10 −68 8	5.24	2632
**Syllablerhyme minus syllableonset**				
Inferior parietal cortex (area 40)	Right	36 −58 58	4.42	472
Occipital cortex (area 17)	Left	−16 −88 −8	4.09	752
Occipital cortex (area 18)	Left	−16 −84 −10	4.12	752

### (1) Semantics

#### Younger adults

When semantics was compared with control matching (Table 1), significant activations were observed bilaterally in the mid-ventrolateral PFC (area 47/12), the mid-dorsolateral PFC (area 9, 9/46), the anterior cingulate cortex (area 32), the posterior parietal cortex (area 7), the occipital cortex (areas 17, 18 and 19), and the cerebellum. The ventrolatrolateral PFC (area 45), the frontopolar cortex (area 10), the lateral premotor cortex (area 6), the posterior cingulate cortex (area 23), and the inferior temporal cortex (area 37, fusiform gyrus) also showed significant activation in the left hemisphere. Subcortically, significant activity was observed bilaterally in the thalamus and the caudate nucleus.

#### Older adults

In the older group (Table 2), there was significant left hemisphere activity in the anterior cingulate cortex (area 32), the mid-dorsolateral PFC (areas 9 and 46), and the SMA (6/8 junction). The cerebellum showed significant right activity.

#### Intergroup comparison

Significant bilateral activation was found in the younger participants versus the older ones in the anterior cingulate cortex (area 32), posterior cingulate cortex (areas 23 and 31), the posterior parietal cortex (area 7), the occipital cortex (areas 17, 18 and 19), and the thalamus. There was also significantly increased activity in the left hemisphere in theventrolateral PFC (area 45), the mid-dorsolateral PFC (area 9), the posterior cingulate cortex (area 23), the lateral posterior parietal cortex (area 7), and the cerebellum (Table 1).

On the other hand, the elderly showed greater bilateral activation in the in the frontopolar cortex (area 10), the insula (areas 41 and 43), the posterior inferior parietal cortex (area 40), and the middle and superior temporal cortices (areas 22 and 39) compared to the young group. There was also significant increased activity in the left hemisphere in the anterior cingulate cortex (areas 32) and the cerebellum, while the posterior cingulate cortex as well as the occipital cortex (area 19) showed increased activation in the right side of the brain (Table 2).

### (2) Syllable onset

#### Younger adults

When syllable onset was compared with control matching in the younger individuals (Table 3), BOLD signal was significantly greater bilaterally in the anterior cingulate cortex (area 32), the ventrolateral PFC (area 47/12), the posterior parietal cortex (area 7), the occipital cortex (areas 17, 18 and 19) and the cerebellum; and significantly greater in the left hemisphere in the frontopolar cortex (area 10), the ventrolateral PFC (area 45), the dorsolateral PFC (area 9, 9/46), the SMA (area 6,8), the lateral premotor cortex (area 6), the posterior parietal cortex (area 40) and the inferior temporal cortex (area 37). Subcortically, significantly increased activity was observed, bilaterally, in the thalamus and the globuspallidus.

#### Older adults

The older group (Table 4) showed bilateral significant activation in the occipital cortex (areas 17, 18 and 19) only. There was, however, also left activation in the mid-dorsolateral prefrontal cortex (areas 9 and 46), the SMA (6/8 junction), and the posterior parietal cortex (areas 7 and 40), as well as right activation in the cerebellum. No significant subcortical activation was observed.

#### Intergroup comparison

The younger group did show significantly increased bilateral activity in the posterior parietal cortex (areas 7 and 40), the precuneus (area 7) and the occipital cortex (areas 17, 18, as well as 19 in the left hemisphere) when compared to the older group. There was also unilateral increased activation in the leftventrolateral PFC (areas 45 and 47/12), the right mid-dorsolateral PFC (area 9), the left SMA (areas 6 and 8), the left posterior cingulate cortex (area 30), the left inferior temporal cortex (area 37) and the left cerebellum (Table 3).

On the other hand, the elderly showed greater bilateral activation in the frontopolar cortex (area 10), and posterior inferior parietal cortex (area 40), the inferior and middle temporal cortices (areas 38 and 39), the posterior cingulate (area 31) and the precuneus (area 7) compared to the young group. There was also significant increased activity in the left hemisphere in the anterior cingulate cortex (areas 32), the lateral premotor cortex (area 6), the insula (areas 41 and 43), the occipital cortex (area 19) and the cerebellum, while the SMA (areas 6 and 8) and the superior temporal cortex (area 22) showed increased activation in the right side of the brain (Table 4).

### (3) Syllable rhyme

#### Younger adults

When syllable rhyme was compared with control matching (Table 5), there was significant bilateral activation in the mid-ventrolateral PFC (area 47/12), the anterior cingulate cortex (area 32), the SMA (area 6, 8), the posterior parietal cortex (area 7), the inferior temporal cortex (area 37, fusiform gyrus), the occipital cortex (areas 17, 18 and 19) and the cerebellum. There was also significant increased activation in the left hemisphere in the frontopolar cortex (area 10), the mid-dorsolateral PFC (area 9), the ventrolateral PFC (area 45), the posterior PFC (area 44), the lateral premotor cortex (area 6), and the posterior parietal cortex (area 40). Finally, significant activation was observed subcortically in the right globuspallidus and bilaterally in the thalamus.

#### Older adults

In the older group (Table 6), there were bilateral significant activations in the SMA (6/8 junction), and the occipital cortex (areas 17, 18 and 19). There was also left increased activity in the mid-dorsolateral PFC (area 9). On the other hand, the right hemisphere sowed increased activity in the cerebellum.

#### Intergroup comparison

The younger group had bilateral significantly increased activity in the occipital cortex (areas 17, 18 and 19) compared to the older one. They also had significant activation in the dorsolateral PFC (area 46), the SMA (6/8 junction), and the posterior parietal cortex (area 40), as well as right activity in the precuneus (area 7) (Table 5).

The elderly, however, showed significant bilateral activity in the lateral premotor cortex (area 6), the insula (areas 41 and 43), the middle temporal cortex (area 39) and the posterior parietal cortex (area 40), right increased activation in the dorsolateral PFC (area 46) and the superior temporal cortex (area 22), as well as increased left activation in the inferior temporal cortex (area 38), the occipital cortex (area 19) and the cerebellum compared with the younger group (Table 6).

### (4) Inter-rules comparisons

#### Younger adults

**Figure 1:**
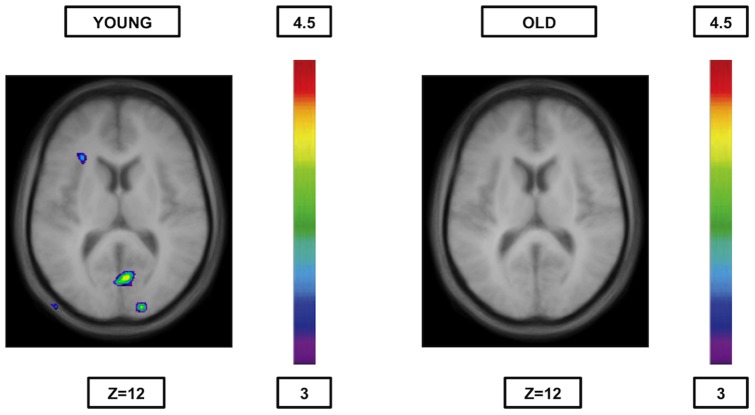
Significant activation when semantics are compared to syllable onset. The younger group (cf. left) shows activation in the left ventrolateral PFC (areas 45 and 47/12), the left temporal regions (areas 37 and 20 - not shown in the figure) and in right occipital regions (areas 17 and 18), while the older group (cf. right) shows no significant peaks of activation at all. The anatomical MRI images are the average of the T1 acquisitions of the 14 younger subjects (cf. left) and the 14 older subjects (cf. right) transformed into stereotaxic space. The color scale represents the T statistic.

**Figure 2:**
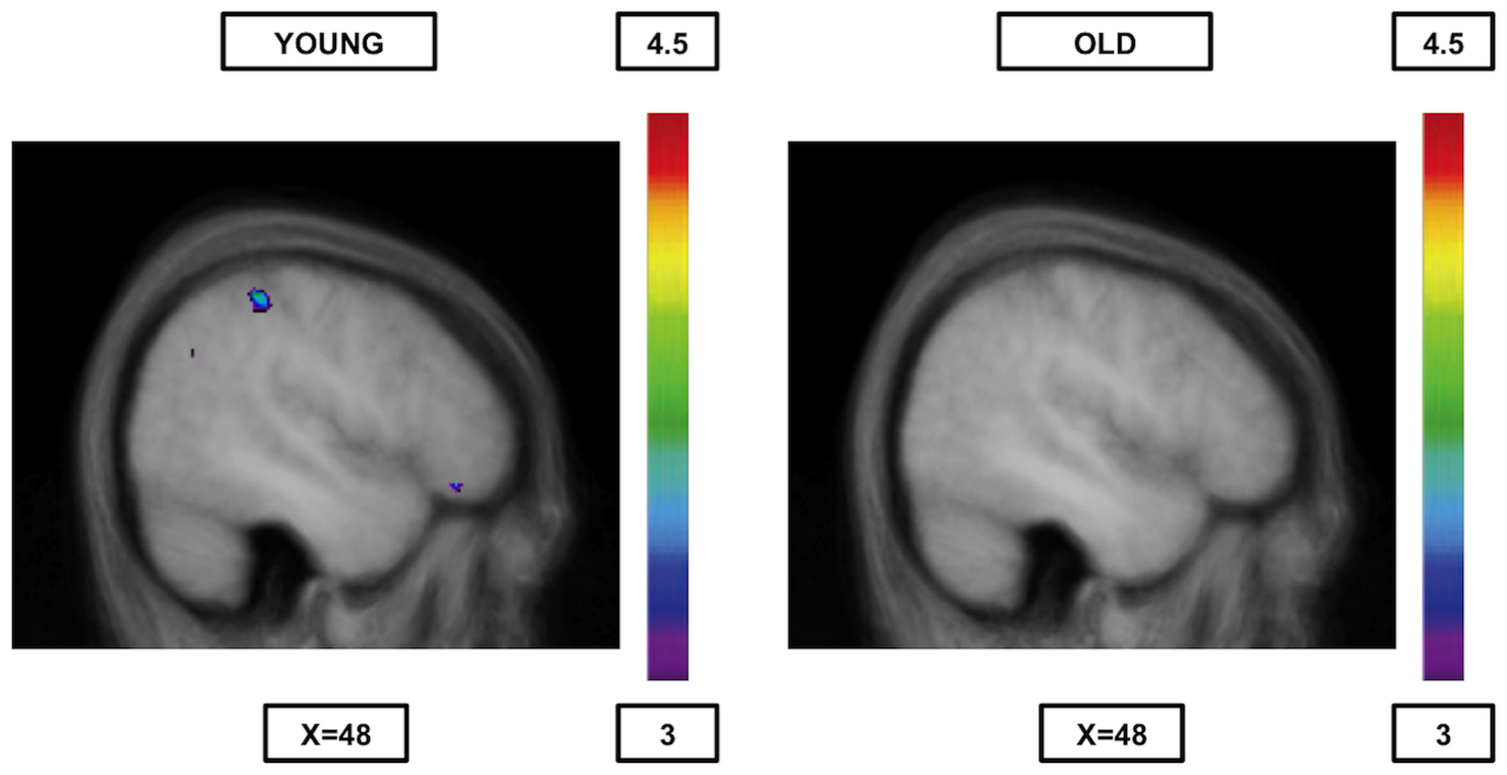
Significant activation when syllable onset is compared to semantics. The younger group (cf. left) shows activation in the right frontopolar area (area 10), the right posterior parietal cortex (area 40), and the left inferior temporal cortex (area 37 – not shown in the figure), while the older group (cff right) shows no significant peaks of activation at all. The anatomical MRI images are the average of the T1 acquisitions of the 14 younger subjects (cf. left) and the 14 older subjects (cf. right) transformed into stereotaxic space. The color scale represents the T statistic.

Comparing BOLD signal during semantics with syllable onset (Table 7, Figure 1) yielded significant activation in the left ventrolateral PFC (areas 45 and 47/12), the left temporal regions (areas 37 and 20) and in right occipital regions (areas 17 and 18). In the reverse comparison (Table 7, Figure 2), syllable onset vs. semantic, there was significant activation in the right frontopolar area (area 10), the right posterior parietal cortex (area 40), and the left inferior temporal cortex (area 37).

When semantics was compared with rhyme (Table 8), significant activations were recorded in the left hemisphere in the ventrolateral PFC (area 45 and 47/12), the dorsolateral PFC (area 9/46), the hippocampus (area 36), the inferior temporal cortex (area 20), and the occipital cortex (area 17), as well as the right caudate nucleus. In the reverse contrast (syllable rhyme minus semantics), significant activation was observed in the left posterior PFC (area 44), left inferior temporal cortex (area 37) and right occipital cortex (area 17) (Table 8).

**Figure 3:**
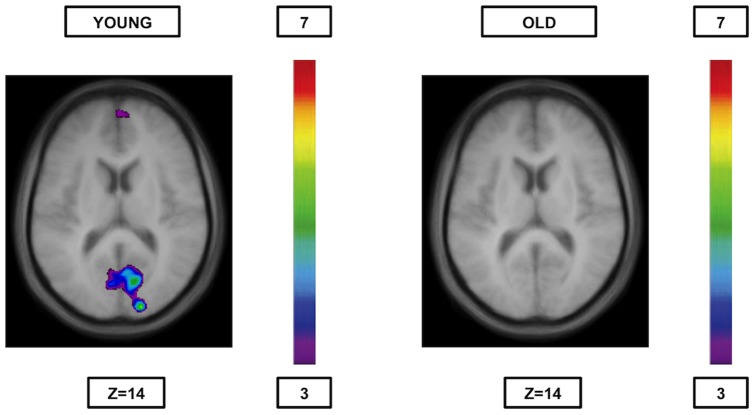
Significant activation when rhyme syllable is compared to syllable onset. The younger group (cf. left) shows significant activation bilaterally in regions 17, 18, and 19 of the occipital cortex and in the right anterior cingulate cortex (area 32), while the older group (cf. right) shows no significant peaks of activation at all. The anatomical MRI images are the average of the T1 acquisitions of the 14 younger subjects (cf. left) and the 14 older subjects (cf. right) transformed into stereotaxic space. The color scale represents the T statistic.

**Figure 4:**
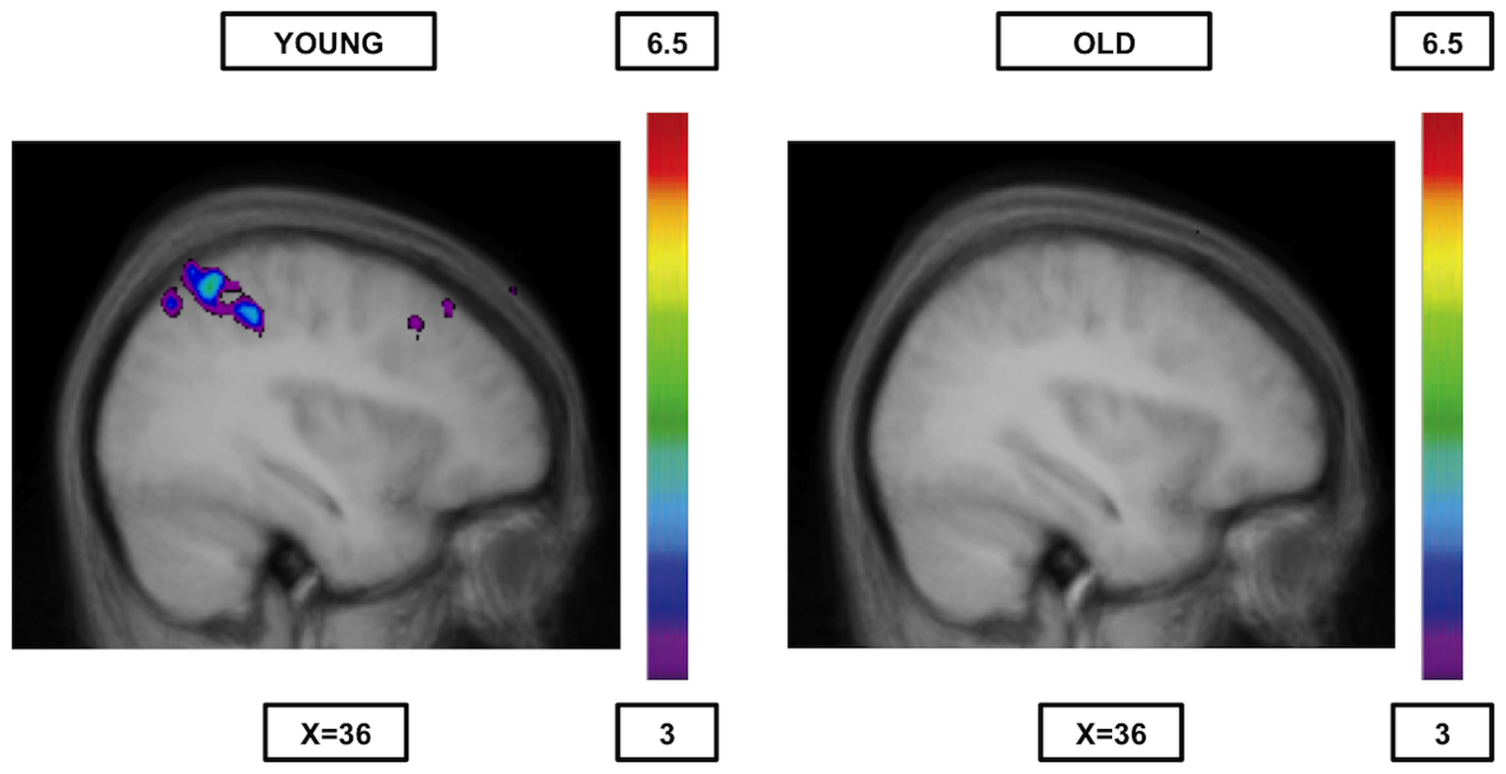
Significant activation when syllable onset is compared to rhyme syllable. The younger group (cf. left) shows significant activation was in the right posterior parietal cortex (area 40) and the left occipital cortex (area 18 - not shown in the figure), while the older group (cf. right) shows no significant peaks of activation at all. The anatomical MRI images are the average of the T1 acquisitions of the 14 younger subjects (cf. left) and the 14 older subjects (cf. right) transformed into stereotaxic space. The color scale represents the T statistic.

When comparing syllable rhyme and syllable onset matching (Table 9, Figure 3) significant activation was observed bilaterally in regions 17, 18, and 19 of the occipital cortex and in the right anterior cingulate cortex (area 32). In the reverse contrast (Table 9, Figure 4), that is syllable onset minus syllable rhyme, significant activation was observed in the right posterior parietal cortex (area 40) and the left occipital cortex (area 18).

#### Older adults

As expected, the comparisons between rules yielded no significant peaks of activation (Tables 7, 8 and 9, Figures 1, 2, 3 and 4).

#### Intergroup comparison

The younger group when compared with the older one showed significant greater right activity in the occipital cortex (areas 17 and 18) when matching according to semantics was compared to syllable onset (Table 7). They also showed significantly increased activity in the left ventrolateral PFC (area 47/12), posterior cingulate cortex (area 23), the inferior temporal cortex (area 20), the inferior parietal cortex (area 40), the precuneus (area 7) and the occipital cortex (area 17) when matching according to semantics was compared to syllable rhyme (Table 8). When syllable onset was compared with syllable rhyme, there was greater right activation in the younger adults compared with the older ones in the mid-dorsolateral PFC (area 9) and the inferior parietal cortex (area 40), as well as left increased activation in the occipital cortex (areas 17 and 18). On the other hand, when syllable rhyme was compared with syllable onset, there was greater right activation in the occipital cortex (areas 17 and 18) (Table 9).

The older group when compared with the younger one had significantly greater activity in the left hemisphere, in the SMA (6 and 8 junction), the posterior cingulate cortex (area 23), theprecuneus (area 7), the occipital cortex (areas 17, 18 and 19) and the cerebellum when matching according to syllable rhyme was compared with matching according to semantics (Table 8). When syllable onset was compared with syllable rhyme, there was greater right activation in the occipital cortex (areas 17 and 18). On the other hand, when syllable rhyme was compared with syllable onset, there was greater activation on the right in the inferior posterior parietal cortex (area 40), and on the left in the occipital cortex (areas 17 and 18) (Table 9).

No significantly greater activity was observed for any other inter-group inter-rule comparison.

## Discussion

As predicted, the results indicate that, with aging, differences between semantic and phonological pathways tend to diminish. Indeed, while younger individuals seem to rely on different regions when performing semantic or phonological functions, older individuals seem to depend on similar routes for both language functions. This observation is in agreement with the recruitment of similar pre-existing brain networks (neural reserve) as well as other brain regions (neural compensation) in order to maintain a high level of performance when demanding tasks are required [30].

Younger individuals showed increased activity in regions belonging to the semantic stream proposed by Devlin [45] and other areas involved in semantic processing [46] [47], namely the dorsolateral PFC, the ventrolateral PFC, the fusiform gyrus, the ventral temporal lobe and the caudate nucleus, plus some other regions more often associated with the phonological (non semantic) pathway [45] (the temporoparietal junction and motor cortical areas) when control matching was subtracted from semantic matching (Table 1). The older group, on the other hand, showed less significant activation for that contrast (Table 2). It is possible that the control condition might have been more difficult for the older group, which led to increased resource recruitment during control matching and therefore less significant activation when the latter was subtracted from matching according to semantics.

A similar pattern was noticed when control matching was subtracted from phonological matching. Indeed, in the younger group, significant activations were found in the left posterior and superior PFC (area 44[for syllable rhyme only], and areas 6 and 8), the inferior temporal cortex (area 37 – involved both in semantic and phonology processing) and the supramarginalgyrus of the posterior parietalcortex (area 40) (Tables 3 and 5). These two areas are known to form the “phonological loop” [45] [48] [49], which is involved in storing and rehearsing verbal information, which is required for verbal working memory [50] [51].It should be noted that other regions such as the frontopolar cortex, the anterior cingulate cortex, the ventrolateral PFC, the dorsolateral PFC and the thalamus were also significantly activated when both phonological rules were compared to controls. These regions were most probably recruited because of the executive demand load required during a set-shifting task (even during non-shifting conditions), indeed these results are similar to those obtained in matching period contrasts in our previous studies using the WCST or the WWST [31] [32] [33] [34]. Interestingly, in the younger group, area 40, together with areas 10 and 37, were the only significantly activated regions when syllable onset matching was compared to semantic matching (Table 7), while area 44, with areas 7 and 37, were the only significantly activated regions when syllable rhyme matching was compared to semantic matching (Table 8), therefore arguing for the importance of areas 37, 40 and 44 in phonological processing. Regarding older individuals, once again, less overall activity was recorded when control matching was subtracted from any of the two phonological rules (Tables 4 and 6) probably because the control matching was more cognitively demanding in this age group as previously mentioned. Moreover, area 44 was not even significantly more activated in any of the phonological rules compared to control matching while area 40 was only significantly activated during syllable onset.

When older individuals were compared to the younger ones, they showed significantly increased activation in the insula (areas 41 and 43), the temporal cortex (areas 22, 38 and/or 39) and the lateral parietal cortex (area 40) for the three classification rules (semantic, syllable onset and syllable rhyme) minus control. These results are in agreement with the fact that older individuals seem to rely on similar pathways when performing both semantic and phonological functions. Indeed they showed, independently of the matching rule, significant activation in areas associated with working memory such as the insula [52], semantic processing such as the temporal cortex [29] [53] and phonological processing such as area 40 [48] [49].

Another interesting finding is the fact that when semantic matching was contrasted with either one of the phonological rules, the younger group showed increased activity mainly in regions within the semantic route (the ventrolateral PFC, the dorsolateral PFC, the inferior temporal cortex and the caudate nucleus[when compared to syllable rhyme only]) plus two other regions not primarily associated with semantic processing, that is the hippocampus (when compared to syllable rhyme only) and the occipital cortex, while the elderly didn't show any increased brain activity at all (Tables 7 and 8; Figure 1). Similarly, when the phonological rules were compared to semantics, there was, in younger individuals, increased activity in areas 37 (both phonological rules), 44 (syllable rhyme) or 40 (syllable onset) as previously mentioned, while the elderly, once again, did not show any increased activity (Tables 7 and 8; Figure 2). This pattern of activation is consistent with previous studies using tasks of phonological perception [54] [55] [56] which showed that area 44 plays an important role in the conversion from orthography to phonology which is more importantly required in the rhyme condition than in the syllable onset condition [32]. Indeed, in the WWST, almost all associations according to the syllable onset condition can be performed by simply comparing word spelling (the letters forming the first syllable). Therefore, there is little need to convert from orthography to phonology in that paradigm. On the other hand, associations according to the syllable rhyme condition rely more heavily on the orthography (spelling) to phonology (sound) conversion (and thus solicitating more area 44). Indeed, words rhyming in “o” can actually finish in “au”, “aut”, “eau”, “o” or “ot”, words rhyming in “e” can end with “é”, “ée” or “er”, and finally words rhyming in “α” can finish in “an”, “eng” or “ent”. Increased activation in the lateral posterior parietal cortex (area 40), on the other hand, was present for both syllable onset and syllable rhyme matching when compared with control matching, this is in agreement with functional imaging studies which noted the activation of area 40 in tasks accessing phonological stores in working memory [57] [58] and requiring phonological processing [59] [60] [61]. Nevertheless, the activation was only recorded in the syllable onset matching when compared to semantics. The reason for this dissimilarity between the two phonological rules remains uncertain, however, it is possible that maintaining in working memory the different word graphologies (which is especially required during the syllable onset condition) may entail more significant involvement of area 40 [32]. It should also be noted that differences in brain activity were found between the two phonological rules for younger individuals, while they were completely absent in the older group (Table 9; Figures 3 and 4). Indeed, the young showed increased activity in the lateral posterior parietal cortex (area 40) when syllable rhyme was subtracted from syllable onset (probably for the same reason mentioned above), and increased occipital and anterior cingulate (area 32) activity in the opposite contrast. It is possible that the syllable rhyme condition requires more attention than the syllable onset condition because of the necessity to convert visual letters forming syllables into sounds in the first condition as previously stated, that would explain why primary and secondary visual regions (occipital cortex) as well as area 32, known to play an important role in focusing attention [62], are significantly more activated during the syllable rhyme matching condition. This being said, the absence of differences between the categorisation rules in the elderly is in agreement with the postulated recruitment of similar global as opposed to specific pathways for semantic or phonological processing in the elderly.Consequently, high-performing old individuals appear to rely on semantic pathways (neural reserve) as well ason other non-semantic language-related regions (neural compensation) during semantic processing, and on phonological pathways (neural reserve) as well as other language (semantic) regions (neural compensation) during phonological processing.

It should be noted that the elderly did show some differences in brain activity in the inter-rule comparisons when they were compared to the young. However, given the nature of intergroup analyses, these results should be interpreted with caution. Therefore, if a region is significantly activated in an intergroup analysis (between groups) for a given contrast, but not in the intragoup analysis for the same contrast (within the group showing the increased activation), the relevance of the significantly increased activity between groups is of limited value. Indeed, it means that for a given contrast (contrast 1), group A likely shows a positive non significant peak of activity in area Z, and that the other group (group B) likely shows a negative peak of activity in the same area Z for the same contrast 1. Thus, when comparing the two groups (A vs B), there is significant activity for contrast 1 in area Z since the negative peak from B, when subtracted from the non significant positive peak from A, gives rise to a more positive (and therefore significant) peak for A minus B. Nevertheless, the difference in activity in area Z between the two conditions forming contrast 1 remains not significant for group A. In the present study, this means that, for the older group, differences between the three conditions are minimal regarding cerebral activation patterns, as previously stated, regardless of the results shown in the intergroup analyses because the latter are largely influenced by negative peaks recorded in the younger group for those same contrasts.

Regarding reaction times, matching periods according to semantics and syllable rhyme were slower than matching periods according to syllable onset in the young. Those results are most probably due to the fact that orthography to phonology conversion was almost not required in the onset syllable condition (as previously stated), but was necessary in the syllable rhyme condition, explaining why matching according to the latter condition took longer than matching according to the first. Matching according to semantics also showed increased response times (compared to syllable onset) because candidates needed to assess semantic categories within the working memory for that condition. Interestingly, in the elderly there were no statistical differences between rule classifications (as it was the case for cerebral activity). The elderly also proved to have slower response times in all classification conditions (except for semantics when control response times were subtracted). This phenomenon is in agreement with an age-related decrease in motor-speed [43] [44].

Finally, we did not observe any age-related intra-hemispheric brain activity reorganization; even if several language studies have shown either increased PFC activity in the elderly [26] or increased posterior activation, especially during semantic processing [27] [28] [29]. On the other hand, the elderly appear to have shown morebilateral activity (HAROLD model) than the young. Indeed, in the intergroup analysis, they presented slightly more bilateral or right activity in the prefrontal, temporal and parietal cortices (Tables 1, 2, 3, 4, 5 and 6). However, the differences were not very important since younger participants also showed significant bilateral involvement. Therefore, in our experiment and as previously argued, neural compensation seems mainly to take the form of recruiting other language processing regions that are usually used for other language processes (rather than bilateralization or intra-hemispheric reorganization of brain activity).

A limitation of the present study is the fairly small sample size of both our groups. Larger groups would have allowed for within group age stratification in order to explore potential differences between “younger” and “older” elderly, since such differences have been found for executive processing [63].Another limitation comes from the fact that we only have one group of older individuals (high performing persons [33]). A third group composed of “low performing” older individuals would have allowed to confirm if all the differences recorded between the elderly and the young were indeed compensatory in nature (and not due to the inability for the elderly to inhibit some none language relevant areas during language processing). This being said, the fact that the older group is a high performing one is in itself an argument for the compensatory nature of the differences in cerebral activity between the two age groups. Furthermore, we might have missed subtle differences between condition rules regarding reaction times. Indeed, for each trial, response times were influenced by how close the matching card was with the curser. Therefore, the number of times a participant had to press on the button (allowing for the curser to move) in order to select the appropriate matching card changed from one trial to another. This increased the reaction time variance within each trial condition, therefore diminishing the ability to find statistical differences in reaction times between conditions. Finally, based on the results obtained in the younger group, there is evidence to show that the two phonological rules of the WWST rely on both similar and different language processes. Indeed, the syllable onset condition appears to require more orthographic than phonological processing, while it appears to be the reverse for the syllable rhyme condition. These dissimilarities between the two phonological rules prevented us from exploring with more precision the effects of aging on “pure” phonological processing, but they did not undermine the principal finding of the study: age-related reduction in language pathways specificity.

## Conclusions

In conclusion, it appears that pathway specificity is reduced with aging. Indeed, in older individuals, the semantic and phonological routes seem to merge into a single one composed of both semantic and phonological pathways. These findings may represent neural reserve/compensation mechanisms in which the elderly, confronted to a demanding lexical task, require to rely more extensively on several brain areas within different language processing routes in order to adequately complete the given task.
